# Pyoderma gangrenosum around an ileostoma: A case report

**DOI:** 10.1097/MD.0000000000013415

**Published:** 2018-11-30

**Authors:** Yong-Ming Yu, Fu-Ji Lai, Chun Feng, Bei-Lei Chen, Yi-Sheng Cao

**Affiliations:** Department of Colorectal Surgery, Ningbo No.2 Hospital, No.41 Northwest Street, Ningbo, Zhejiang Province, China.

**Keywords:** ileostomy, peristoaml dermatitis, pyoderma gangrenosum

## Abstract

**Rationale:**

Pyoderma gangrenosum (PG) is a rare postoperative complication of enterostomy, mostly developing from dermatitis, which may have serious consequence.

**Patient concerns:**

A patient with lower rectal cancer receiving low anterior resection (LAR) and protective ileostomy was initially diagnosed with dermatitis, which very quickly developed to PG, though no medical or familial history was found.

**Diagnosis:**

We diagnosed the patient with peristoaml dermatitis starting from a tiny skin ulceration, but corrected the diagnosis to PG because of the rapid development and severe consequences.

**Interventions:**

Routine stoma care did not improve the condition, so we performed 2 terms of debridement, the closure of the stoma and autologous skin transplantation before finally solving the problem.

**Outcomes:**

The patient was discharged 60 days after the first surgery and 5 days after the last one. After 18 months of follow-up, the patient kept in a stable condition.

**Lessons:**

Medical staff should not neglect peristoaml dermatitis because of its common occurrence. Once the situation develops beyond the doctors′ expectation, more efforts should be made to treat it, even expand debridement if possible.

## 1 Introduction

Protective ileostomy is widely performed for both lower rectal cancer patients receiving low anterior resection (LAR) and severe inflammatory bowel disease (IBD) receiving total colectomy to prevent anastomotic leakage.^[1]^ Common stoma related complications include ileostomy prolapse, stomal retraction, electrolyte imbalance, peristomal hernia, and peristomal dermatitis.^[2]^ Most of these complications manifest mildly and can be cured by definite diagnosis and early treatment. Recently, we confronted a severe dermatitis around the ileostoma which ultimately developed to pyoderma gangrenosum (PG). PG, whose clinical appearance is characterized by sudden onset of sterile pustules that rapidly develop into painful ulcerations, and undermined borders, is often associated with systemic diseases such as IBD, rheumatoid arthritis, diabetes, neoplasms, or metabolic syndrome in over 50% of cases.^[3]^ In our case, PG was very similar to peristomal dermatitis at the very beginning from a tiny skin ulceration but progressed so rapidly beyond doctors′ expectation.

## 2 Case report

A 51-year-old man presented with hematochezia, thin and frequent defecation for 2 months before being admitted to the hospital. Colonoscopy showed a cauliflower-like mass in the rectum 5 cm above the anus, occupying 4/5 of the rectal circle. The biopsy pathology suggested “adenocarcinoma”. The patient had no medical or familial history. He had smoked 20 cigarettes daily for 30 years and drank white wine 100 g daily for 2 years. He was admitted with normal vital signs and a BMI (Body Mass Index) of 20.6 kg/m^2^. Digital palpation found a hard mass 5 × 5 cm in size and slight intestinal stenosis. Routine blood, biochemical test, blood gas analysis, and blood coagulation function found no obvious abnormality. His CEA level was 2.34 ng/mL and CA19–9 10.27 U/ml. Chest high-resolution computed tomography (HRCT) found a localized emphysema in bilateral lungs. Abdominal enhanced computed tomography (CT) revealed non-homogeneous thickening and enhancement of the rectal wall in the middle and lower segment with no enlarged peripheral mesenteric lymph nodes or distant metastases.

The patient was diagnosed as cT4aN0M0 rectal cancer. Multiple disciplinary team (MDT) was held to discuss whether neoadjuvant therapy should be performed. In the view of incomplete intestinal obstruction, the patient′s suffering and his insistence to receive operation as soon as possible, laparoscopic radical resection of rectal cancer (Dixon) and protective end ileostomy was carried out 4 days after his admission. The operation was successful with only 10 ml of blood loss recorded during the procedure. The patient returned to ground activity in 24 h, exhausted and defecated from the orifice in 48 h. His temperature remained within normal range; his white blood cell (WBC) and C-reactive protein returned to normal in 5 days. The incision, as well as the stoma and its surrounding skin, healed well 3 days after operation.

**Figure 1:**
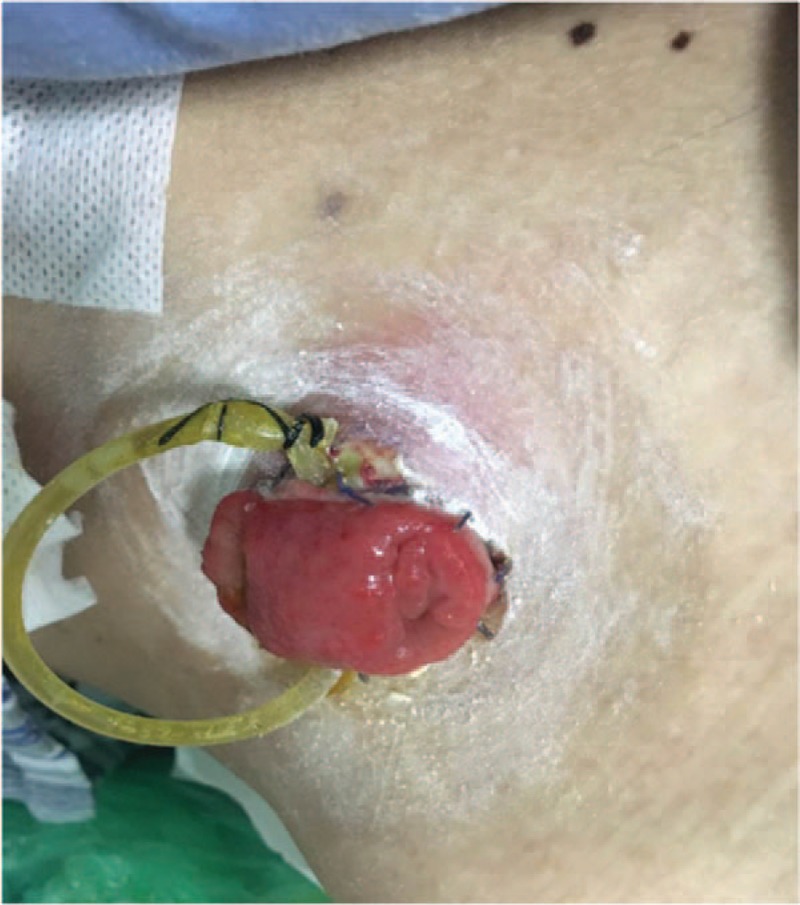
A tiny skin irritation on the upper side of stoma where the supporting plastic loop was located.

**Figure 2:**
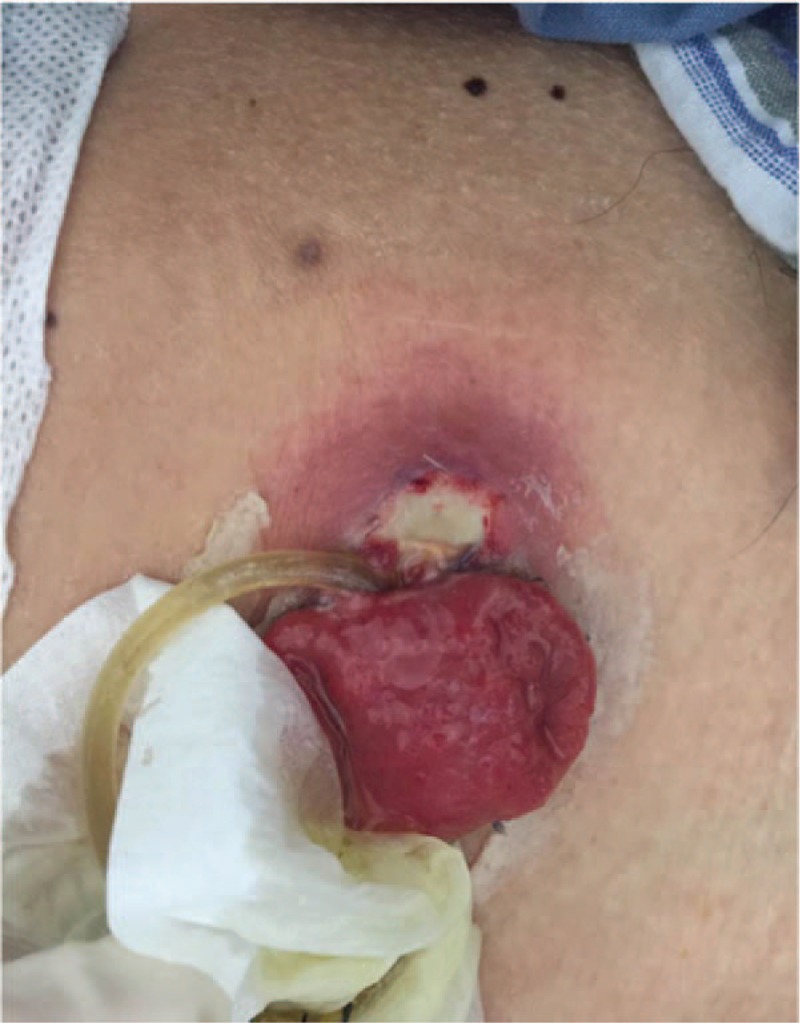
Red and edema skin with deep ulcer and pus moss at upper and lower side of the stoma.

**Figure 3:**
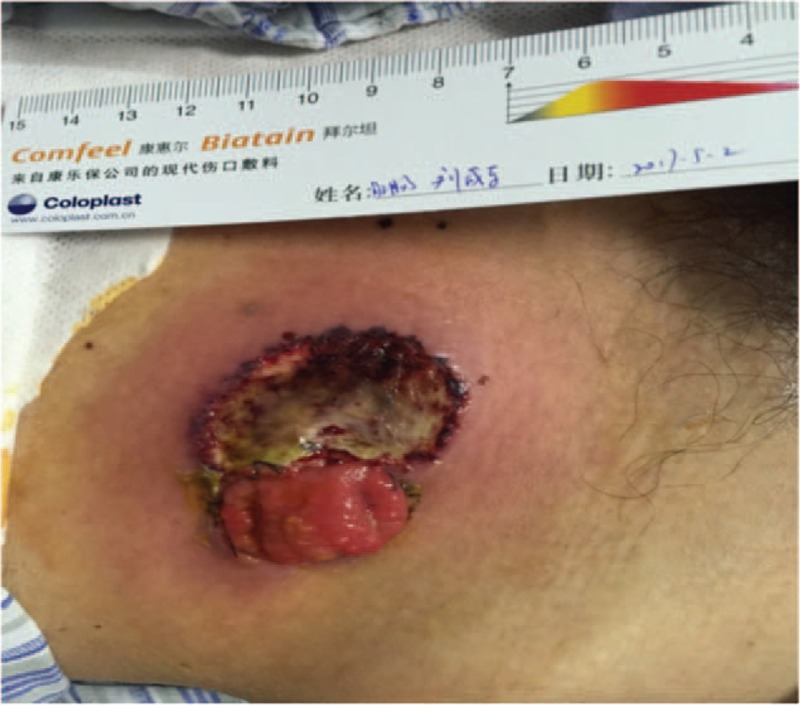
The upper side wound with an ulceration area of 4 cm in diameter.

**Figure 4:**
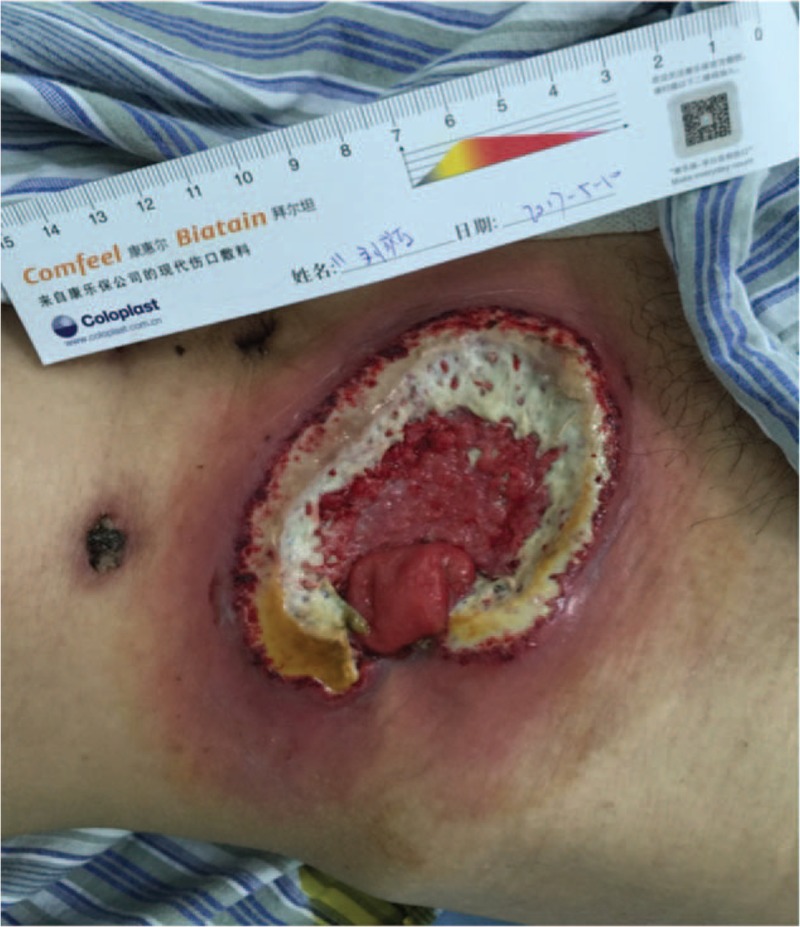
The ulceration taking up almost the whole circle around the orifice.

**Figure 5:**
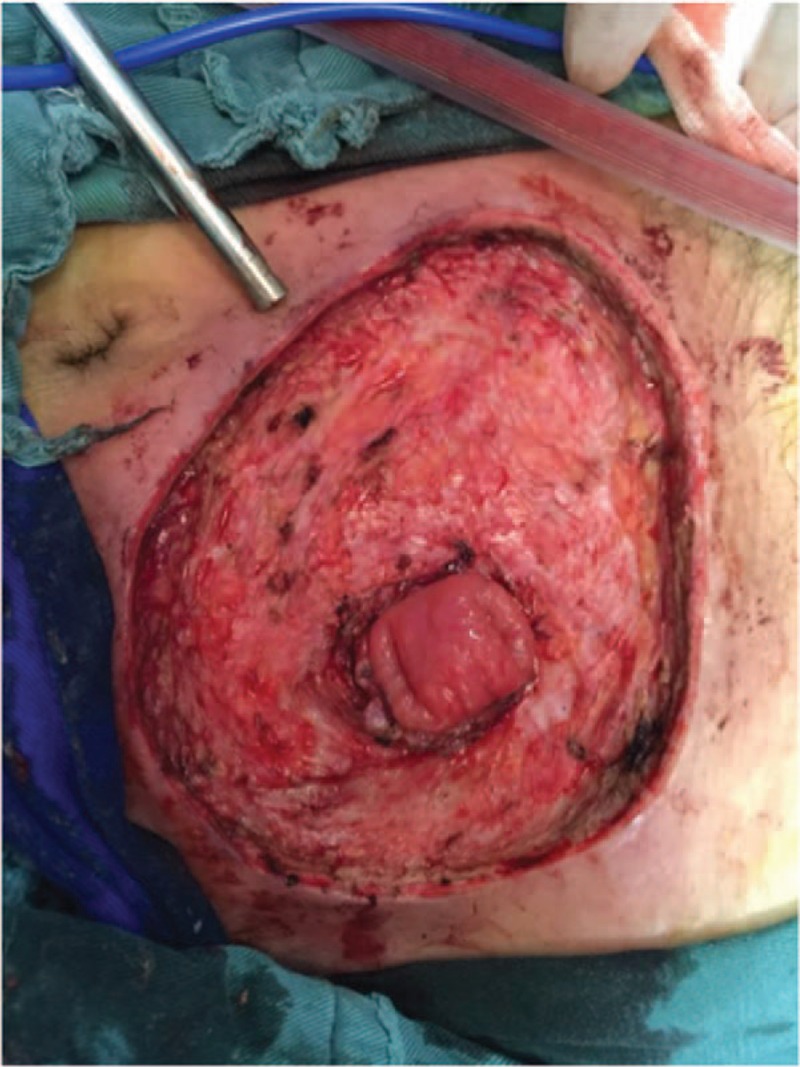
The infective tissue was completely resected deep into the fascia superficialis layer.

**Figure 6:**
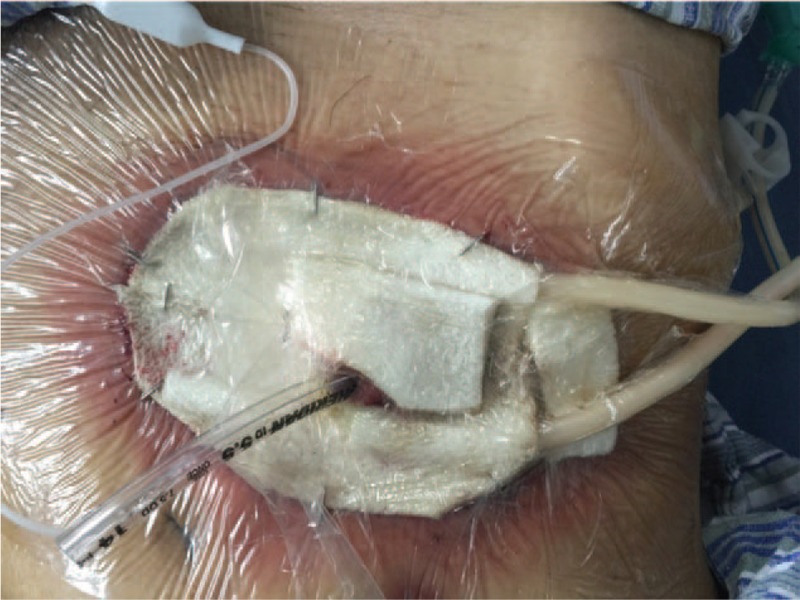
The wound was paved with VSD. VSD = vacuum sealing draina.

**Figure 7:**
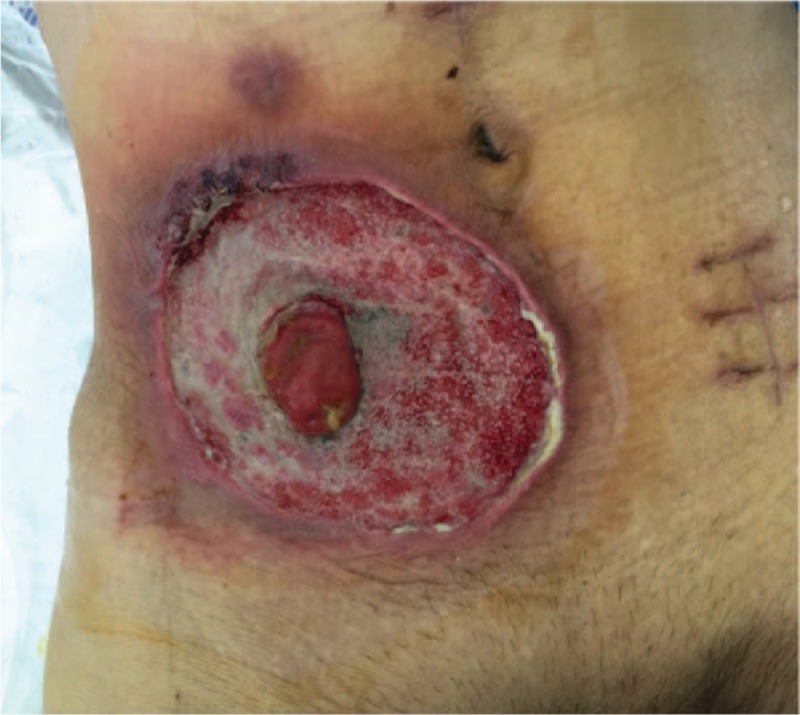
A small area of pus moss came out again with necrosis on the upper right side.

**Figure 8:**
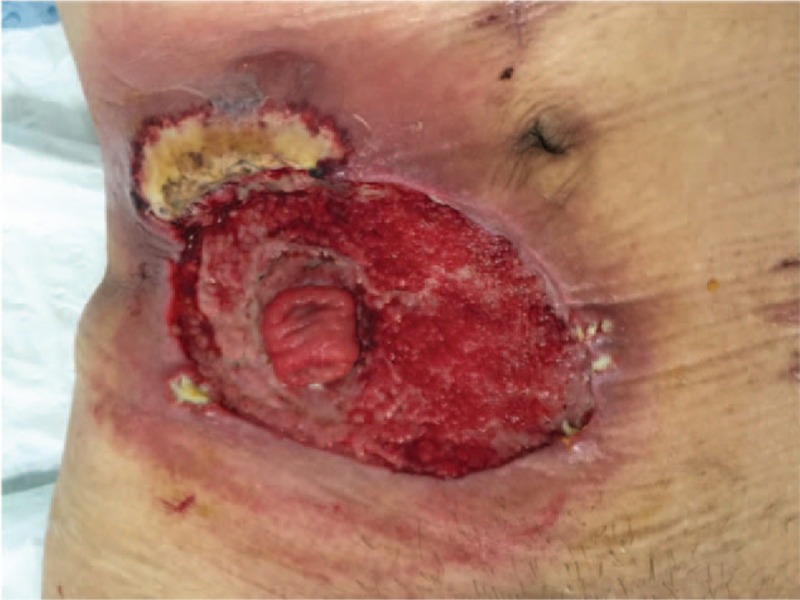
Pus moss again covered the upper right side of the wound.

**Figure 9:**
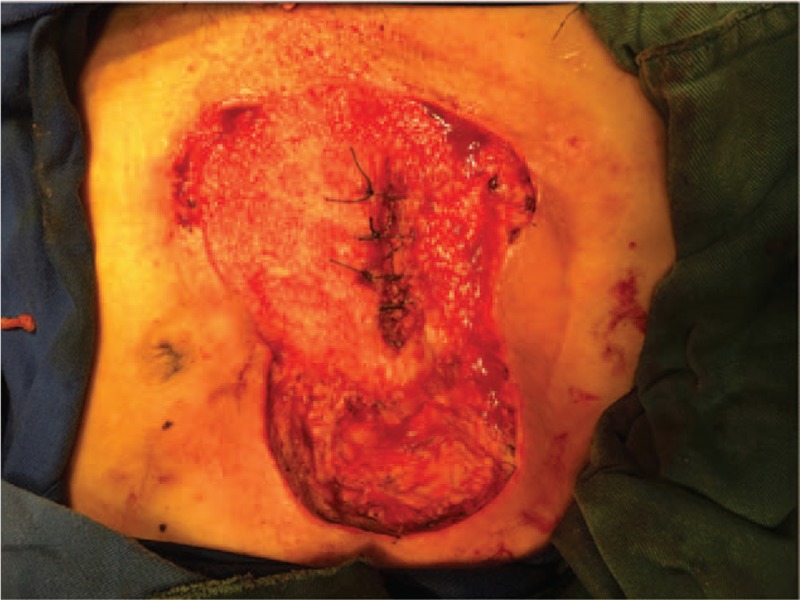
The ileostoma was closed and the infected abdominal wall was debrided again.

**Figure 10:**
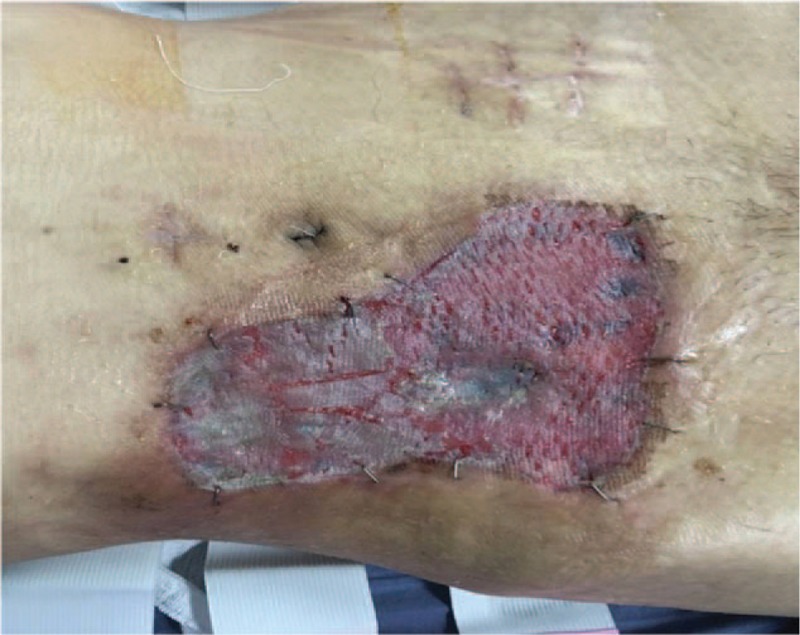
The wound was healed after autologous skin transplantation.

On day 7, the drain pipe was removed after CT scan showing no evidence of leakage or infection. On day 8, routine blood test showed the WBC count jumped to 20.1 × 10^9^/L, with neutrophil ratio up to 82.5%; no fever or pain occurred. Only a tiny skin irritation on the upper side of stoma was found, where the supporting plastic loop was located (Fig. 1). The patient was diagnosed with peristomal dermatitis. The loop was adjusted, and the ostomy bag model was changed (from Alterna 02833 + 01698 Coloplast into 05985 Coloplast) in order to increase the frequency of stoma observation. No obvious abnormality in nutritional status or electrolyte balance occurred. On day 10, both upper and lower side of the stoma saw red and edema skin with deep ulcer and pus (Fig. 2). The supporting loop was removed immediately and pus was cleared until fresh tissue emerged. The patient was told to take food with less liquid and montmorillonite powder was also prescribed to make the fecal less watery. At daytime, the stoma was kept uncovered and cleaned as long as any fecal fluid outflew from the orifice. Ostomy bag was only used at night to guarantee adequate sleep time. Zinc oxide ointment, Coloplast Brava 1907 skin care powder and Prep Barrior Film 62041 were also used to isolate the skin from the irritant. The upper side wound ulcerated to an area of 4 cm in diameter but the lower one healed 6 days later (Fig. 3). Besides, the skin 1–2 cm peripheral to the margin of the ulceration showed a color of dark red. A catheter was inserted to the proximal end of the ileostomy, and then connected to the vacuum aspiration with continuous low negative pressure. The temperature of the patient remained normal and the pain from the wound was slight except when we cleared the necrotic tissue. WBC count declined to 12.0 × 10^9^/L, with neutrophil ratio 80.7% after mezlocillin was used for 7 days. One more week later the ulcer area increased, taking up almost the whole area around the orifice (Fig. 4). A lot of pus moss covered the surface and obvious purulent drainage exuded from the wound edge. Debridement of the involved abdominal wall was performed under general anesthesia. Infective tissue surrounding the ileostoma was completely resected deep into the fascia superficialis layer, paving with vacuum sealing draina (VSD) (Figs. 5 and 6). On day 37, when the VSD was removed, the wound came to us with fresh granulation tissue as well as some purulent secretion. The lower left side of the wound saw a small area of pus moss again, and necrosis in the upper right side (Fig. 7). On day 41, the same situation happened as we had seen before (Fig. 8). So the burn surgery specialist suggested closure of the stoma before doing dermatoplasty. Considering it had been 40 days since LAR and the patient recovered well except for the peri-stoma dermatitis, we did an operation to close the ileostoma and debride the infected abdominal wall again under general anesthesia (Fig. 9). After that, the wound healed rapidly without any signs of infection. Burn surgery specialist then performed harvesting grafts from the scalp and abdominal autologous skin transplantation on day 55. The patient was discharged 60 days after the first surgery and 5 days after the last one (Fig. 10). After 18 months of follow-up, the skin graft healed well without any infection, and the tumor did not recur or metastasize.

## 3 Discussion

PG is an uncommon, ulcerative cutaneous condition of uncertain etiology, typically presenting with single or multiple skin ulcers with undetermined erythematous-violaceous borders, and it may precede, coexist, or follow the different systemic diseases, such as IBD.^[4,5]^ Treatment of PG consists of suppression of inflammatory disease activity, treatment of associated morbidities, promotion of wound healing, as well as pain relief.^[3]^ In this case, PG was brought on by the development of peristomal dermatitis, which was a common stoma related complication, frequently seen from patients with ileostomy because of the watery fecal and irritation of small intestinal juice.^[6]^ The pH value of intestinal juice is about 7.8, and it contains a large amount of digestive enzymes and bile salts, which can easily result in chemical injury, especially when the skin has a wound. When it happens, irritants should be isolated from the surface with powder or barrior film to prevent further infection. Some enterostomal therapists even insert a catheter to the proximal end of the ileostomy with continuous low negative pressure to stop the juice coming out from the stoma and contaminating the wound.^[7]^ Another potential cause is the ostomy bag which leads to the change of the micro-environment of the skin. Mechanical pressure, impermeable surface and bacterial translocation make peristomal skin more vulnerable to ulceration and infection. Immune disorders also come from the primary diseases which will in turn affect the susceptibility of the skin. In most cases, dermatitis will be alleviated after careful nursing of the stoma, so it is under-recorded and unrecognized.^[8]^

Peristomal dermatitis is more frequent in women, and the high incidence population locates at the age between 20 and 50 years;^[9]^ 70% of cases occur related to ileostomies.^[10]^ The case we reported was severe and rare, which progressed so rapidly and resisted any treatment. There is no optimal way of treatment so far because patients’ condition differs a lot.^[11]^ Both local and systemic factors should be considered during the whole treatment. More attention needs to be paid to the nutritional status, immune function and electrolyte balance of the patient, especially when IBD is the primary disease. In our case, we treated this patient as a common dermatitis sufferer at the beginning, but the effect was poor. When we realized the severity from the fast-spreading skin lesion, nothing could have stopped its development. After the first debridement, the wound seemed to be clean and fresh, but there was still purulent secretion days later, which meant the infection spread from the ileostomy into the layer deeper than fascia superficialis. The closure of the stoma cut down the source of the irritant and finally cured the patient. But if the stoma could not be closed for now, relocation would be the only resolution. In our opinion, peristomal dermatitis and PG are not 2 independent diseases, but 2 stages of disease development. Doctors should take it seriously. When peristomal dermatitis happens, if not recognized in time or treated properly, PG may subsequently occur in the patient.

## Acknowledgments

The authors will thank burn surgery specialist Chun Zhang for his great help in treatment of this patient.

## Author contributions

**Conceptualization:** Yi-Sheng Cao.

**Data curation:** Yong-Ming Yu and Yi-Sheng Cao.

**Formal analysis:** Yong-Ming Yu.

**Investigation:** Fu-Ji Lai, Bei-Lei Chen, and Yi-Sheng Cao.

**Project administration:** Fu-Ji Lai and Yi-Sheng Cao.

**Resources:** Chun Feng and Bei-Lei Chen.

**Supervision:** Yong-Ming Yu, Chun Feng, and Bei-Lei Chen.

**Visualization:** Fu-Ji Lai, Chun Feng, and Bei-Lei Chen.

**Writing – original draft:** Yi-Sheng Cao.

**Writing – review & editing:** Yi-Sheng Cao.

Yi-Sheng Cao orcid: 0000-0002-8195-6826.
